# The Mode of Action of Spatial Repellents and Their Impact on Vectorial Capacity of *Anopheles gambiae sensu stricto*

**DOI:** 10.1371/journal.pone.0110433

**Published:** 2014-12-08

**Authors:** Sheila B. Ogoma, Hassan Ngonyani, Emmanuel T. Simfukwe, Antony Mseka, Jason Moore, Marta F. Maia, Sarah J. Moore, Lena M. Lorenz

**Affiliations:** 1 Environmental Health and Ecological Sciences, Ifakara Health Institute, Bagamoyo, Tanzania; 2 Infectious and Tropical Diseases, London School of Hygiene and Tropical Medicine, London, United Kingdom; 3 Department of Epidemiology and Public Health, Swiss Tropical & Public Health Institute, Basel, Switzerland; 4 University of Basel, Petersplatz, Basel, Switzerland; New Mexico State University, United States of America

## Abstract

Malaria vector control relies on toxicity of insecticides used in long lasting insecticide treated nets and indoor residual spraying. This is despite evidence that sub–lethal insecticides reduce human–vector contact and malaria transmission. The impact of sub–lethal insecticides on host seeking and blood feeding of mosquitoes was measured. Taxis boxes distinguished between repellency and attraction inhibition of mosquitoes by measuring response of mosquitoes towards or away from Transfluthrin coils and humans. Protective effective distance of coils and long-term effects on blood feeding were measured in the semi–field tunnel and in a Peet Grady chamber. Laboratory reared pyrethroid susceptible *Anopheles gambiae sensu stricto* mosquitoes were used. In the taxis boxes, a higher proportion of mosquitoes (67%–82%) were activated and flew towards the human in the presence of Transfluthrin coils. Coils did not hinder attraction of mosquitoes to the human. In the semi–field Tunnel, coils placed 0.3 m from the human reduced feeding by 86% (95% CI [0.66; 0.95]) when used as a “bubble” compared to 65% (95% CI [0.51; 0.76]) when used as a “point source”. Mosquitoes exposed to coils inside a Peet Grady chamber were delayed from feeding normally for 12 hours but there was no effect on free flying and caged mosquitoes exposed in the semi–field tunnel. These findings indicate that airborne pyrethroids minimize human–vector contact through reduced and delayed blood feeding. This information is useful for the development of target product profiles of spatial repellent products that can be used to complement mainstream malaria vector control tools.

## Introduction

The probability of mosquito vectors successfully transmitting disease pathogens to a host depends on their ability to effectively locate the host and blood feed. Among factors that influence the rate at which new human malaria infections are disseminated per day by a mosquito i.e. vectorial capacity, is the man – biting rate of mosquitoes [1]. Man – biting rate describes the frequency of mosquitoes to bite humans. For malaria parasites to be transmitted from one person to another, mosquitoes need to blood feed at least twice: firstly to ingest parasites and then secondly to infect another human. [2]. Even though man – biting rate is just one component of the vectorial capacity, it profoundly influences malaria transmission and substantially contributes to variation in the stability of malaria transmission and is critical where vectors are anthropophilic, i.e. prefer feeding on humans [3].

Efficient malaria vectors including: *Anopheles gambiae sensu stricto (s.s.)* and *An. funestus s.s* have evolved innate host seeking and feeding preferences for humans due to their ability to discern human kairomones from other hosts [4]. These mosquitoes locate and orient towards hosts at distances [5], [6] as far as 30 meters [7]. Several human odours have been identified as olfactory cues that govern mosquito host seeking and feeding behavior [8], [9]. Studies of the insect olfactory system have led to identification and development of synthetic chemical compounds that attract insects to hosts [10]. This knowledge is successfully applied in the agricultural sector for the control of crop pests [11] and tsetse flies [12], [13], [14] as well as the control of mosquitoes [15], [16], [17].

Other volatile compounds, commonly known as repellents, interfere with mosquitoes' host finding ability. They are intended to reduce human – mosquito contact and have been shown to reduce disease transmission [18]. Repellency has been described as: 1) “taxis” – immediate directional movement of target insects such as mosquitoes, away from the source of the chemical and; 2) “orthokinesis” – increased mosquito activity after contact with insecticides [19], [20]. Other studies indicate that volatile compounds such as DEET, linalool, dehydrolinalool, catnip oil and citronella interfere with the attraction of mosquitoes to host odors by blocking natural responses to attractants, hence acting as attraction inhibitors and not repellents [21], [22], [23]. Lucas *et al* (2007) suggested that even in the presence of airborne pyrethroids, mosquitoes were able to detect host odors but were inhibited from feeding: “When mosquitoes detected host odors, flew upwind and landed, the majority of insects were still inhibited from biting. This effect is probably a result of pyrethroid – induced neural hyperexcitation, that can occur at much lower doses than those required for insect knockdown and mortality” [24].

Mosquito behavior elicited in response to airborne compounds including movement away from a chemical stimulus, loss of host detection, anti-feeding as well as knockdown and mortality are collectively referred to as spatial repellency. Spatial repellents do not require physical contact of the mosquito with treated surfaces like insecticides used in indoor residual spraying (IRS) and long lasting insecticidal nets (LLINs), but act in the vapour state at a distance. Mosquito coils, candles and emanators impregnated with volatile pyrethroids and other compounds such as plant terpines are collectively known as spatial repellents. Among these products, coils have been extensively studied [25] and are commonly used to control mosquitoes [26]. Coils prevent mosquitoes from entering houses, induce early exit and reduce human biting [27], [28]. Despite numerous evaluations of coils, their mode of action is not clear: Do they interfere with orientation of mosquitoes towards humans, inhibit blood feeding or even induce both processes? It is essential to ascertain which of these actions is at play in order to aid the development of effective spatial repellents. This study aimed to distinguish between repellency as described by Dethier [20] and attraction inhibition [29] induced by airborne pyrethroids using the taxis box system [7] to measure the orientation of *An. gambiae s.s*. mosquitoes towards and away from humans in the presence of airborne pyrethroids. The protective distance conferred by coils was measured by the immediate reduction of blood feeding mosquitoes on human volunteers conducting landing catches in the semi – field tunnel (SFT). In addition, the long-term effects of exposure to coils on mosquitoes were investigated by measuring the length of time beyond which the blood feeding inhibition state was extended after mosquitoes were exposed to different doses of pyrethroids coils.

## Materials and Methods

### Test compounds

Mosquito coils contained different doses of Transfluthrin including: 0.015%, 0.03% and 0.045% as well as blank coils that do not have any active ingredient.

### Mosquitoes

Laboratory reared pyrethroid susceptible *An. gambiae s.s.* Ifakara strain mosquitoes were used. During rearing, larvae were fed on Tetramin fish food while adults were fed on human blood between 3 and 6 days after emergence and offered 10% glucose solution *ad libitum*. Temperature within the insectary was maintained between 28–29°C, between 70–80% relative humidity and natural light periods (12:12 hours light: dark periods). Female nulliparous 3–8 days old mosquitoes that had never blood fed and sugar starved for 6 hours prior to starting experiments were used for all studies.

## Experiment 1: Orientation of Mosquitoes in the Presence of Coils and Humans

### Taxis boxes system

A new assay using taxis boxes to measure long-range mosquito responses to different stimuli developed at IHI [7] was used to measure effect of coils on the orientation of mosquitoes towards humans. Briefly, taxis boxes consist of three chambers measuring 40×40×40 cm separated by metal sheets: one chamber facing the stimuli, the middle chamber and one chamber facing away from the stimuli. The middle chamber has a “letter box slit” (30 cm long and 2.5 cm wide) on either side that allows mosquitoes to leave the middle but reduces the likelihood of them returning. During the experimental period, the metal sheets were lifted using a simple pulley mechanism that opened the slits and allowed mosquitoes to fly through. The pulley comprised a rope and lever located 10 meters from the boxes. The boxes were raised 15 cm from the floor and the wooden stands were placed inside plastic cups that contained water and grease. This prevented ants from reaching the mosquitoes.

### Experimental design

**Figure 1:**
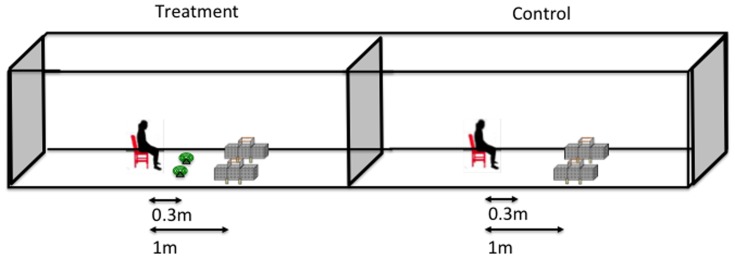
Taxis boxes experimental design. Two taxis boxes placed 1 m away from the stimulus/treatment that is: a human or human and a coil. Mosquitoes were introduced in the middle chamber of each taxis box and the stimulus/treatment was changed each day to determine the effect on orientation of mosquitoes.

A fully randomized study was conducted. The study involved 6 treatments; 1) a positive control – human without a coil; 2) human + blank coil; 3) human +0.015% coil; 4) human +0.03% coil; 5) human +0.045% coil and 6) a negative control – no human and no coil. The last treatment was included in order to measure mosquito response in the absence of any stimulus. The taxis boxes were placed 1 m away from the treatment (Figure 1). A treatment was randomly allocated to an experimental night using the lottery method. The treatments were tested four times using four human volunteers randomly assigned on a nightly basis to give an average human response. Two taxis boxes were used to increase the sample, but each box was treated as a separate factor in the analysis to ensure independence of experimental replicates.

### Procedure

Experiments were conducted between 1830 and 2200 hours. Wind speed and direction were measured nightly using a hand-held anemometer (Heavy weather WS - 2300 or WS – 2310). Wind speed was between 0 and 2 meters/minute. The netting on the walls of the tunnel allowed occasional gusts of air to pass through. Thirty female mosquitoes were placed in the middle chamber of each taxis box and left to acclimatize for 20 minutes. The metal sheets were pulled up and left open for 2 hours to allow mosquitoes to respond to the stimulus. The following morning, mosquitoes were collected from the chambers using mouth aspirators.

## Experiment 2: Protective Distance of Coils against Outdoor Biting Mosquitoes

### Semi-field tunnel (SFT)

The SFT is 100 meters long by 3 meters wide. The walls and roof of the tunnel are screened with fiberglass netting supported by metal frames. A palm-thatch roof approximately 1 m above the netting roof protects the tunnel from direct sunlight and rain. The tunnel was operated at temperatures of 24°C–29°C at night.

### Experimental design

#### a. Point source experiments

**Figure 2:**
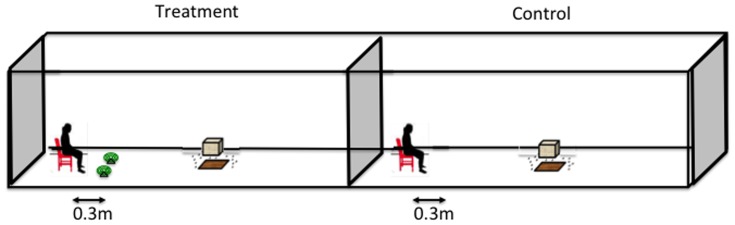
Point source experimental set up. These experiments were conducted in the semi – field tunnel. In the control, two coils were placed on one side of the human. The distance between the coils and the human was changed each day to determine the protective distance of coils. Coils were not used in the control. Mosquitoes were released inside the tunnel and they were left to acclimatize for 10 minutes and then the human started collecting mosquitoes that landed on the bare feet.

A partially randomized study was conducted inside the SFT. Treatments included 1) control (human alone) and 2) treatment (two 0.03% Transfluthrin coils next to a human). The SFT was divided into two equal compartments, each measuring 30 m×2 m×1.5 m. A plastic sheet between the compartments prevented airflow between them. On the first night of experiments, treatments and two volunteers were randomly allocated to each compartment. This was followed by a pairwise rotation of volunteers and treatments between compartments on consecutive experimental nights. The control was always conducted first in the chosen compartment followed by the treatment in the other compartment after 2 hours on the same night. In the treatment, two 0.03% Transfluthrin coils were placed at a specified distance from the volunteer, hence creating a single source from which the chemical was released (Figure 2). This arrangement is referred to as “point source”. The protective distance of coils was evaluated by placing two coils at six different distances from the human at 0.3 m, 1 m, 5 m, 10 m, 15 m, 20 m and 30 m. These distances were randomly allocated to each experimental night and each distance was repeated four times.

#### b. Bubble experiments

**Figure 3:**
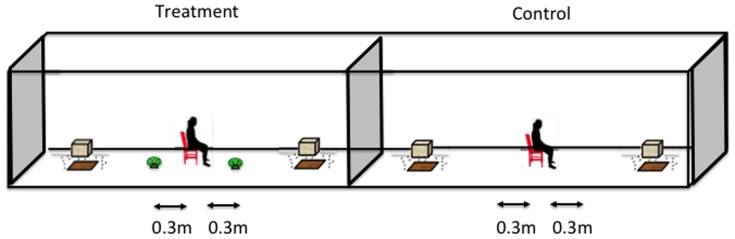
Bubble experimental set up. Experiments were conducted in the semi – field tunnel. A coil was placed equidistant on either side of the human. The distance was changed each night to determine the protective distance. Coils were not used in the control. Mosquitoes were released inside the tunnel and left to acclimatize for 10 minutes and then the human started collecting mosquitoes.

A partially randomized study was conducted. Treatments included 1) control (human alone) and 2) treatment (two 0.03% Transfluthrin coils next to a human). The same two volunteers from the “point source” experiment also conducted the “bubble” experiment. Experiments were conducted in a 60 m long compartment. Unlike the point source, the treatment and control were tested on separate days in order to minimize contamination of the control experiment with insecticide residues from burning coils. Treatments were allocated to day one and day two and a volunteer was allocated to each night. Volunteers were switched between nights such that at the end of 4 days both volunteers had been paired with the control and treatment once, which resulted into a four – day block. Six distances (0.3 m, 1 m, 5 m, 10 m, 15 m, 20 m and 30 m) were randomly allocated to each four – day block. In this set up, one coil was placed equidistant on the left hand side and another coil on the right hand side of the volunteer at the designated distance creating a “bubble” of chemical around the volunteer (Figure 3).

Experiments were started at 1830 hours each evening. One hundred female *An. gambiae s.s*. aged between 3 and 8 days and previously starved for 6 hours were released from cages placed inside the tunnel by a pulley system (Figure 3) and operated from outside the tunnel. Mosquitoes were left to acclimatize for 20 minutes and a volunteer entered the tunnel. Volunteers collected mosquitoes that landed on the bare legs and feet for 2 hours using mouth aspirators. Mosquitoes were kept in labeled paper cups for counting the following morning. All mosquitoes were kept in the testing room whose temperature was maintained between 28–29°C and 70–80% relative humidity.

## Experiment 3: Resumption to Blood Feeding of Mosquitoes after Exposure to Coils

### Peet Grady chamber experiments

#### Experimental design

A fully randomized study was conducted. Treatments included: 1) a negative control (no coil) 2) blank coil; 3) 0.015% coil; 4) 0.03% coil and 5) 0.045% coil. These treatments were randomly assigned to five days of experiments in a 5×5 Latin square design. One hundred female mosquitoes exposed to a treatment were randomly divided into equal batches of 10 mosquitoes per cup. Two cups of mosquitoes were randomly assigned to each blood feeding time regime, namely 15 minutes, 1 hour, 12 hours and 24 hours blood feeding after exposure to coils. Each treatment was repeated five times.

#### Procedure

The Peet Grady chamber [30] was fitted with a battery operated fan to provide ventilation. One hundred female mosquitoes were placed in 30 cm by 30 cm netting cages at 1830 hours. A treatment was applied (e.g. a 0.03% Transfluthrin coil was lit) inside the chamber and after 10 minutes, the cage containing mosquitoes was placed inside on a stool. Mosquitoes were exposed to the burning coil for two minutes and then they were transferred to the laboratory and the coil was extinguished. Mosquitoes were kept in a testing room with temperature maintained between 28–29°C and between 70–80% relative humidity. Mosquitoes were gently aspirated and placed into paper cups labeled with the allotted blood feeding time. Pieces of cotton wool soaked in 10% glucose solution were placed on the remaining paper cups. The cotton wool was removed six hours prior to each specific feeding time. After each time interval had elapsed, a human arm was placed above the paper cups and mosquitoes were allowed to feed through the netting for 15 minutes. The number of fed and unfed mosquitoes in each cup was counted and recorded. Experiments with the control were conducted in the same way except that mosquitoes were not exposed to a coil.

### Semi – field tunnel experiments

#### Experimental design

The experiment included two treatments in the SFT; 1) control (no coil) and 2) treatment (two 0.03% Transfluthrin coils). Treatments were randomly allocated to two days of experiments and one treatment was tested each day. Female mosquitoes were simultaneously exposed to the treatments in the SFT in two different ways; 1) caged mosquitoes and 2) free flying mosquitoes. Experiments were conducted in a 20-meter long SFT lined with white plastic sheets to enable easy location of mosquitoes that were knocked down. Both treatments were repeated four times.

#### Procedure

Experiments were started at 1830 hours. In the caged mosquitoes set up, 25 female mosquitoes were each placed in four 30 cm by 30 cm netting cages. Cages were suspended inside the tunnel one meter above the floor approximately half a meter apart from each other and from two burning 0.03% Transfluthrin coils placed on the floor. In the free flying mosquitoes set up, 100 female mosquitoes were placed in a 30 cm by 30 cm netting cage. The cage was placed in the middle of the chamber. A pulley was operated outside the tunnel to release mosquitoes to fly freely inside the tunnel. For both assays, mosquitoes were left in the tunnel for two hours after which caged mosquitoes were removed and free flying mosquitoes were recaptured using mouth aspirators. Knocked down and dead mosquitoes were collected from the floor. All mosquitoes were kept in the testing room whose temperature was maintained between 28–29°C and 70–80% relative humidity. Live mosquitoes were placed into paper cups. Two paper cups were allocated to each blood feeding time regime. The time regimes were 1 hour, 12 hours, 18 hours, and 24 hours after mosquitoes had been exposed to burning coils or the control. Mosquitoes were blood fed at the allocated time by placing an arm above the cup for 15 minutes and the number of fed and unfed mosquitoes was recorded. Pieces of cotton wool soaked in 10% glucose solution were placed on paper cups to maintain mosquitoes in between blood feeding. The glucose pads were removed six hours prior to blood feeding.

## Protection of Participants and Ethical Approval

The volunteers were recruited on a voluntary basis through written informed consent. The risks and benefits of the study were clearly explained, and they were free to leave at any time during the study. Volunteers were provided with clothing that protected them from the cold temperature at night and were advised to dress in shorts that reached the knees with covered shoes to avoid bites on the feet. They were required not to smoke, take alcohol or use scented soaps and deodorants six hours prior to experiments. The participants were screened for malaria at the beginning of the study and those found with malaria were given Artemisinin Combination Therapy antimalarial drugs and referred to the nearest health center. Those fit to participate in the study were tested for malaria every two weeks. Adverse events such as respiratory symptoms were monitored. The participants were also compensated for their time and effort. The ethical review boards of Ifakara Health Institute IHI/IRB/No A-019-2007, the National Malaria Research Institute Tanzania (NIMR/HQ/R.8a/Vol.1X/710) and the London School of Hygiene and Tropical Medicine (LSHTM ERB 5552) approved the study.

## Statistical Analysis

Data was analyzed using the R statistical software version 2.15.0 [31] with significance level of 0.05 for rejecting the null hypothesis. All generalized linear mixed models (GLMMs) were conducted using the lme4 package [32].

### Experiment 1: Orientation of mosquitoes in the presence of coils and humans

#### a. Activation of mosquitoes to the stimulus

It was assumed that the distribution of mosquitoes in the taxis boxes is a result of movement in response to stimuli. We set the proportion of mosquitoes that were activated by the stimuli equal to the proportion of mosquitoes that left the middle chamber. This was determined by dividing the total number of mosquitoes in the away and towards chamber by the total number of mosquitoes in the taxis boxes including those in the middle chamber. Generalized mixed effects models with binomial error structure and logit link function were used to analyze the behavior of mosquitoes in taxis boxes. The dependent variable was the proportion of activated mosquitoes. Independent variables included treatment and taxis box code as fixed factors and day as a random factor.

#### b. Attraction of mosquitoes to the stimuli

Mosquitoes that were collected from the chamber towards the stimuli were considered to be attracted to the stimulus. Therefore, the proportion of attracted mosquitoes was determined by dividing the number of mosquitoes found in the chamber towards the stimuli by the total number of mosquitoes in the taxis box. Attraction of mosquitoes was analyzed using a GLMM with binomial error structure and logit link function. The dependent variable was the proportion of attracted mosquitoes. The independent variables were treatment and taxis box code as fixed factors and day as a random factor.

#### c. Repellency of mosquitoes by the stimuli

Mosquitoes found in the chamber away from the stimuli were considered to be repelled. This was determined by dividing the number of mosquitoes in the away chamber by total number of mosquitoes in the taxis boxes. Mosquitoes repelled were analyzed using a GLMM with binomial error structure and logit link function. The dependent variable was the proportion of repelled mosquitoes. The independent variables were treatment and taxis box code as fixed factors and day as a random factor.

### Experiment 2: Protective distance of coils against outdoor biting mosquitoes

Data from the point source and bubble experiments were analyzed separately. GLMMs were used to determine the proportion of biting mosquitoes at different distances with reference to the control. The dependent variable was the proportion of blood fed mosquitoes while the independent variables included treatment (control and coil), distance and their interaction, which were fixed categorical variables. The day of experiment was included as a random variable. The models were fitted with a binomial error and a logit link function.

### Experiment 3: Resumption to blood feeding of mosquitoes after exposure to coils

The data from the Peet Grady, caged and free flying experiments were analyzed separately. GLMMs were fitted with a binomial error and a logit link function. The dependent variable was the proportion of blood fed mosquitoes. Treatment, time regime at which mosquitoes were offered blood and their interaction were set as fixed categorical variables and day of experiment as a random variable.

## Results

### Experiment 1: Orientation of mosquitoes in the presence of coils and humans

#### a. Activation of mosquitoes

**Figure 4:**
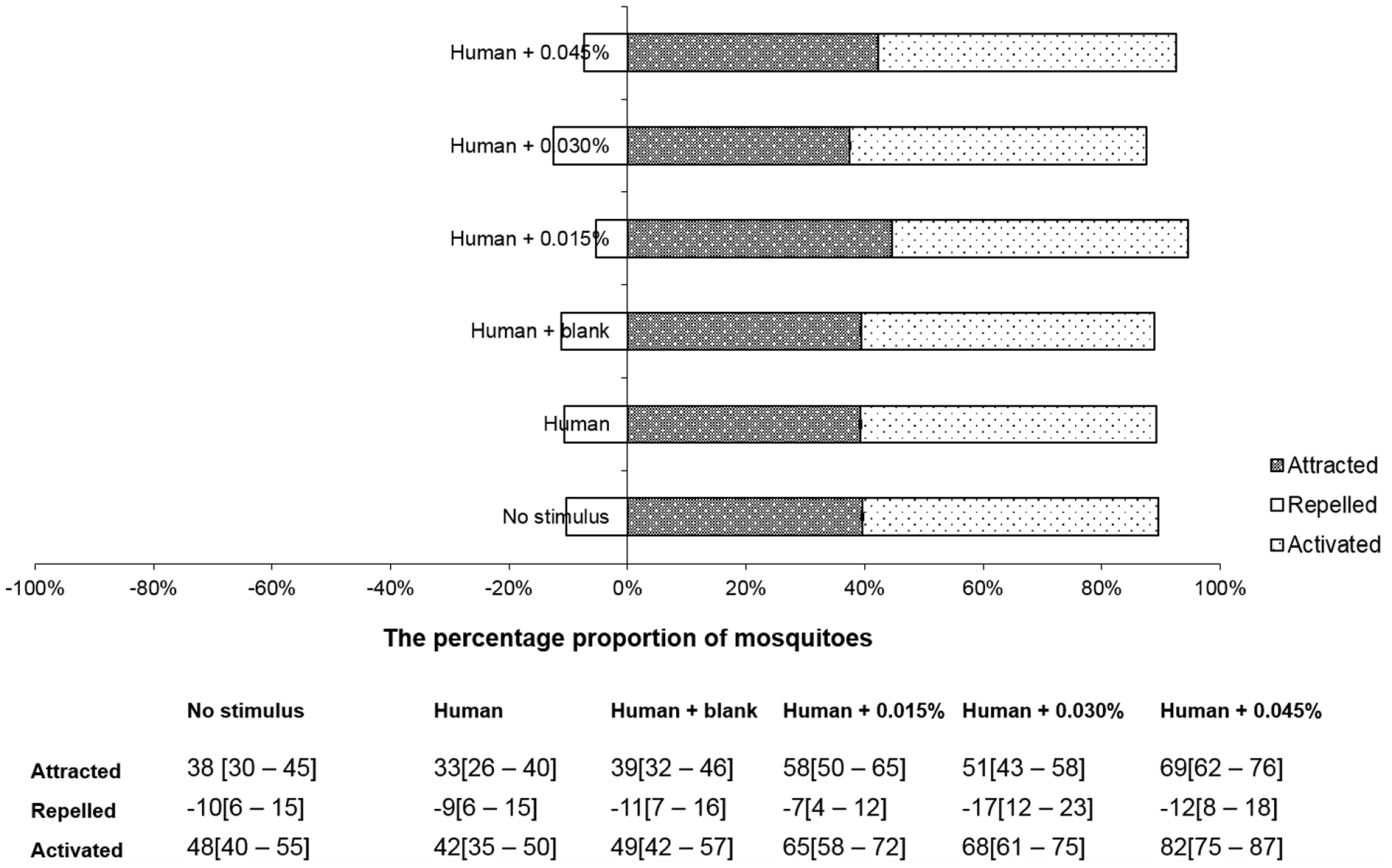
Dose response of mosquitoes to Transfluthrin coils with a human using taxis boxes. Horizontal histogram presenting the percentage proportion of mosquitoes activated, attracted and repelled by control (no stimulus) and treatments: human alone, human + blank coil, human +0.015% Transfluthrin coil, human +0.030% Transfluthrin coil, human +0.045% Transfluthrin coil) in the taxis boxes. The table includes percentage proportions and their confidence intervals.

The proportion of activated mosquitoes increased with increasing Transfluthrin dose (Figure 4). About 82% of the mosquitoes left the middle chamber when 0.045% coils were placed next to the human. The activation of mosquitoes by all the three doses of Transfluthrin was significantly higher compared to the proportion of mosquitoes activated where there was a human alone (Table 1). The proportion of activated mosquitoes was lowest (42%–49%) when there was no Transfluthrin.

**Table 1:** The odds ratios and proportion of activated and attracted mosquitoes in taxis boxes placed 1 meter away from different doses of mosquito coils and a human.

Treatment	Odds ratio Activated^a^ [95% CI]	Proportion Activated^a^ [95% CI]	z Value	p Value	Odds ratio Attracted^b^ [95% CI]	Proportion Attracted^b^ [95% CI]	z Value	p Value
**Human**	1.00 [0.57–2.18]	0.42 [0.30–0.54]	-	-	1.00 [0.70–1.74]	0.32 [0.23–0.42]	-	-
**Human + blank**	1.34 [0.87–3.27]	0.49 [0.32–0.66]	0.824	0.410	1.30 [0.97–2.43]	0.38 [0.25–0.53]	0.917	0.359
**Human +0.015%**	2.58 [1.62–6.66]	0.67 [0.49–0.80]	2.845	0.004	2.85 [2.08–5.41]	0.58 [0.43–0.72]	3.639	0.001
**Human +0.030%**	2.93 [1.90–7.30]	0.68 [0.51–0.81]	3.034	0.002	2.12 [1.57–3.95]	0.50 [0.36–0.65]	2.531	0.011
**Human +0.045%**	6.13 [3.95–15.92]	0.82 [0.69–0.91]	4.988	0.001	4.65 [3.43–8.81]	0.69 [0.55–0.81]	5.160	0.001
**No stimulus^c^**	1.25 [0.80–3.13]	0.48 [0.31–0.65]	0.735	0.462	1.25 [0.92–2.34]	0.38 [0.25–0.52]	0.822	0.411

CI – Confidence intervals; ^a^ - Model estimated mean proportions of activated mosquitoes. The proportion of activated mosquitoes was calculated by dividing the number of mosquitoes collected from the chambers of taxis boxes facing towards and away from the treatment by mosquitoes collected from all chambers of the taxis box. ^b^ - Model estimated mean proportions of attracted mosquitoes. The proportion of attracted mosquitoes was calculated by dividing the number of mosquitoes collected from the chambers of taxis boxes facing towards the treatment by mosquitoes collected from all chambers of the taxis box. ^c^ – There was no human or coil, representing movement of mosquitoes in response to nature.

#### b. Attraction and repellency of mosquitoes

Approximately half of the mosquitoes were attracted when 0.015% and 0.03% Transfluthrin coils were used (Figure 4 and Table 1). The highest dose of Transfluthrin (0.045%) induced a significantly higher proportion of attracted mosquitoes (69%) relative to the human alone (33%) (z = 5.160; p = 0.001) (Table 1).

The proportion of repelled mosquitoes ranged between 7% and 17% (Figure 4) and was not significantly different from the human alone (human + blank coil: z = 0.296; p = 0.767, human +0.0015%: z = −0.656; p = 0.572, human +0.03%: z = 1.895; p = 0.058, human +0.045%; z = 0.789; p = 0.430, human alone: z = 0.185; p = 0.853). This indicates that the taxis boxes did not detect movement of mosquitoes away from coils and humans.

### Experiment 2: Protective distance of coils against outdoor biting mosquitoes

#### a. Coils placed on one side of the human: ‘point source’

Smoke from Transfluthrin coils prevented mosquitoes from effectively locating hosts with fewer mosquitoes landing in the presence of coils. Coils were most effective when placed 0.3 m away from volunteers. Approximately 20% (95% CI [0.12; 0.31]) of the mosquitoes fed when the coil was 0.3 m away compared to 65% (95% CI (0.51; 0.76) when there was no coil (z = 12.206; p = <0.001) (Table 2). The proportion of feeding mosquitoes also decreased when coils were placed between 1 m and 20 m, but there was no significant reduction of blood feeding mosquitoes when coils were placed 30 m away (Table 2).

**Table 2:** The proportion of biting mosquitoes in the presence of 0.03% Transfluthrin coils placed as a point source at different distances.

Distance	Control	Treatment	95% CI	z Value	p Value	Odds ratio
**0.3 m**	257/400(0.65)	80/400(0.20)	[0.12–0.31]	−12.206	<0.001	0.14
**1 m**	167/400(0.41)	88/400(0.21)	[0.12–0.31]	−6.153	<0.001	0.39
**5 m**	177/400(0.44)	114/400(0.28)	[0.18–0.41]	−4.709	<0.001	0.50
**10 m**	394/400(0.90)	274/440(0.63)	[0.49–0.75]	−9.017	<0.001	0.19
**15 m**	344/440(0.79)	252/440(0.57)	[0.43–0.71]	−6.595	<0.001	0.37
**20 m**	347/440(0.80)	273/400(0.63)	[0.48–0.75]	5.535	<0.001	0.44
**30 m**	147/400(0.33)	156/400(0.36)	[0.24–0.50]	0.713	0.476	1.10

CI-Confidence intervals; the values placed in brackets in the control and treatment columns are model estimated mean proportions.

#### b. Coils placed on the left and right side of the human: ‘bubble’

Coils were most effective when they were placed 0.3 m away from the human. Approximately 4% (95% CI [0.01; 0.13]) of the mosquitoes fed when the coil was 0.3 m away compared 86% (95% CI [0.66; 0.95] when there was no coil (z = −5.546; p<0.001) (Table 3). The odds of mosquitoes landing on a human next to a coil increased slightly as the distance between the coils and the human increased (Table 3). There was no significant difference in the proportion of landing mosquitoes when coils were placed 30 m away (Table 3).

**Table 3:** The proportion of biting mosquitoes in the presence of 0.03% Transfluthrin coils creating a ‘bubble’ around the user.

Distance	Control	Treatment	95% CI	z Value	p Value	Odds ratio
**0.3 m**	80/100(0.86)	4/100(0.04)	[0.01–0.13]	−5.546	<0.001	0.01
**1 m**	259/600(0.43)	12/600(0.02)	[0.01–0.04]	−11.950	<0.001	0.02
**5 m**	331/600(0.41)	5/800(0.01)	[0.00–0.01]	−10.580	<0.001	0.009
**10 m**	216/600(0.35)	8/600(0.01)	[0.01–0.03]	−10.210	<0.001	0.02
**15 m**	83/100(0.84)	39/100(0.37)	[0.17–0.63]	−2.808	0.005	0.13
**20 m**	70/100(0.71)	37/100(0.37)	[0.17–0.62]	−1.891	0.060	0.25
**30 m**	90/100(0.92)	78/100(0.79)	[0.56–0.92]	−1.353	0.176	0.39

The values placed in brackets in the control and treatment columns are model estimated mean proportions, CI Confidence intervals of the means.

### Experiment 3: Resumption to blood feeding of mosquitoes after exposure to coils

#### a. Peet Grady chamber experiments

**Figure 5:**
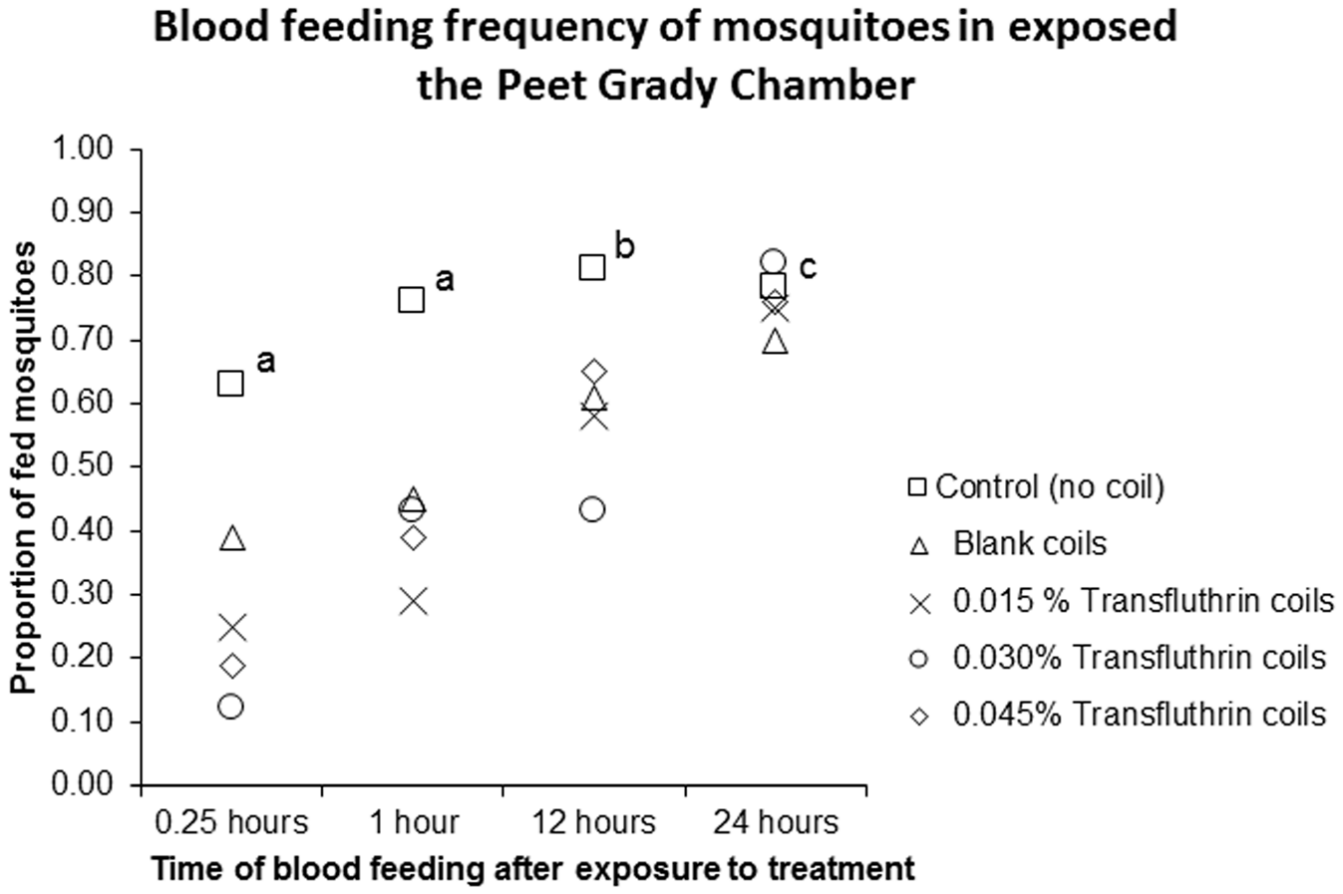
The effect of Transfluthrin coils on blood feeding behavior of mosquitoes in a Peet Grady chamber. Mosquitoes were exposed to different doses of Transfluthrin coils inside a Peet Grady chamber and later offered blood meals at different time intervals. The proportion of blood fed mosquitoes was compared between different doses and the control that had no coil. The proportion of blood fed mosquitoes was significantly lower than the control in all treatments after 25 minutes (^a^) and 1 hour (^a^). At 12 hours only 0.03% Transfluthrin coils significantly (^b^) reduced feeding compared to the control while after 24 hours there was no significant difference between all treatments and controls (^c^).

The proportion of fed mosquitoes was lowest at 12% (95% CI [0.06; 0.22]), (z = −5.301; p<0.001) 15 minutes after exposure to 0.03% Transfluthrin coils. The presence of smoke without the insecticide (blank coil) significantly inhibited feeding after 15 minutes (Table 4) but the proportion of mosquitoes inhibited from feeding was lower than when Transfluthrin coils were used. The effect of Transfluthrin coils demonstrated a dose response relationship although increasing the dose beyond 0.03% had little effect (Figure 5).

**Table 4:** The proportion of mosquitoes that blood fed at different time intervals following exposure to different doses of Transfluthrin coils inside a Peet Grady chamber.

Time (Hours)	Treatment	Fed/Total	Mean proportion a	95% CI	z Value	p Value	Odds ratio
**0.25**	**Control**	61/98	0.63	[0.49–0.76]	-	-	1.00
	**Blank**	42/100	0.39	[0.27–0.53]	−2.409	0.174	0.53
	**0.015%**	23/99	0.25	[0.15–0.38]	−3.815	0.002	0.18
	**0.030%**	12/99	0.12	[0.06–0.22]	−5.301	<0.001	0.08
	**0.045%**	20/100	0.19	[0.11–0.31]	−4.393	<0.001	0.08
**1**	**Control**	74/100	0.76	[0.62–0.85]	-	-	1.00
	**Blank**	47/98	0.45	[0.32–0.59]	−3.115	0.025	0.32
	**0.015%**	26/98	0.29	[0.18–0.42]	−4.674	<0.001	0.13
	**0.03%**	43/100	0.43	[0.29–0.54]	−3.267	0.015	0.13
	**0.045%**	39/99	0.39	[0.26–0.53]	−3.610	0.005	0.23
**12**	**Control**	78/98	0.81	[0.69–0.89]	-	-	1.00
	**Blank**	58/93	0.61	[0.47–0.73]	−2.254	0.245	0.42
	**0.015%**	55/100	0.58	[0.45–0.71]	−2.544	0.126	0.31
	**0.030%**	41/94	0.43	[0.30–0.58]	−3.810	0.002	0.20
	**0.045%**	60/93	0.65	[0.50–0.77]	−1.842	0.516	0.47
**24**	**Control**	71/93	0.78	[0.65–0.87]	-	-	1.00
	**Blank**	66/93	0.70	[0.57–0.81]	−0.915	0.992	0.76
	**0.015%**	69/96	0.75	[0.63–0.84]	−0.379	1.000	0.79
	**0.03%**	71/88	0.82	[0.69–0.90]	0.443	1.000	1.29
	**0.045%**	64/85	0.76	[0.62–0.86]	−0.273	1.000	0.94

a – Model estimated mean proportions, CI Confidence intervals of means.

Exposure to burning coils also influenced subsequent blood feeding. The proportion of mosquitoes that took blood up to 12 hours after exposure to 0.03% and 0.045% Transfluthrin coils were significantly lower compared to the control (Table 4). In addition, the propensity of mosquitoes to feed increased gradually with time irrespective of whether they were exposed to Transfluthrin coils or not. Results indicate that at some point between 12 and 24 hours, there was no difference in the proportion of fed mosquitoes between the control and coils (Table 4), showing that mosquitoes resume normal feeding one day after indoor exposure to Transfluthrin coils.

#### b. Semi – field tunnel experiments

**Figure 6:**
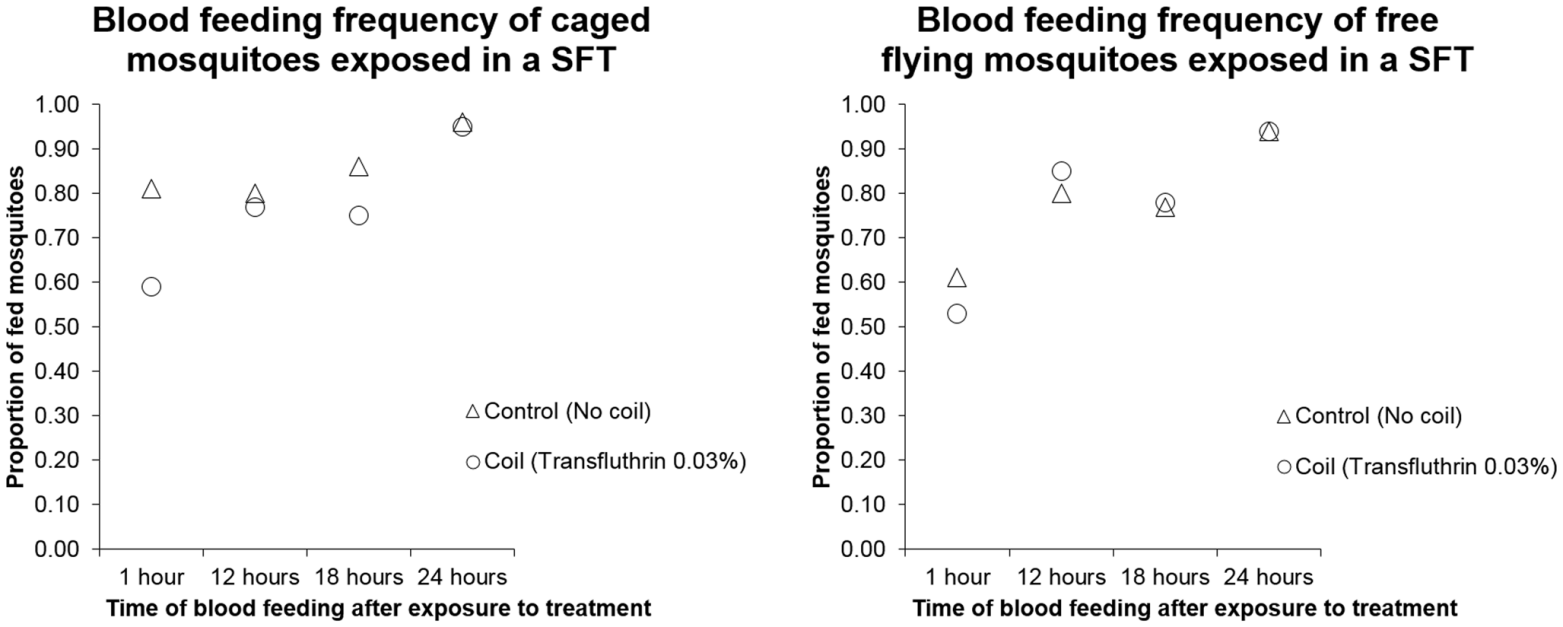
The effect of Transfluthrin coils on blood feeding behavior of mosquitoes in a Semi-Field Tunnel. The proportion of blood fed mosquitoes after they had been exposed to mosquito coils in the semi-field tunnel is presented in the two graphs. The graph on the left indicates caged mosquitoes and the one on the right indicates free flying mosquitoes. In the left graph the proportion of blood fed mosquitoes was significantly lower in the treatment compared to the control after only after 1 hour. In the right graph, the proportion of blood fed mosquitoes was not significantly different between controls and treatments at all times.

Two Transfluthrin coils (0.03%) did not influence the feeding behavior of free flying mosquitoes exposed outdoors in the netting tunnel. The proportion of mosquitoes that fed after exposure to coils was not significantly different from the control (z = −0.943; p = 0.346); around 53% (42/76) (95% CI [0.43; 0.67]) blood fed after 1 hour after exposure to Transfluthrin compared to 61% (48/80) (95% CI [0.48; 0.71]) in the control. More than three quarters of the mosquitoes had fed after 12 hours and subsequent time feeding intervals (Figure 6). There was no significant difference between the proportion of fed mosquitoes in the control and the treatment at subsequent feeding times (12 hours: z = 0.526; p = 0.599, 18 hours: z = −0.169; p = 0.866, 24 hours: z = −0.098; p = 0.922).

There was a slight impact on the feeding behavior of caged mosquitoes (Figure 6). After 1 hour, 56% (35/62) (95% CI [0.43; 0.69]) mosquitoes exposed to Transfluthrin fed compared to 79% (61/77) (95% CI [0.69; 0.88]) in the control (z = −2.937; p = 0.003) and after 18 hours 84% (59/70) (95% CI [0.74; 0.92]) mosquitoes had blood fed compared to 72% (39/54) (95% CI [0.58; 0.84]) in the treatment (z = −2.445; p = 0.015). However, more than three quarters of the mosquitoes fed after 12 hours and 24 hours and this was not significantly different between the control and the treatment (12 hours: z = −1.341; p = 0.180, 24 hours: z = −0.006; p = 0.996).

## Discussion

This study highlights challenges in the measurement of mosquito responses to different stimuli whilst outdoors. Mosquito activity such as orientation towards humans, oviposition and resting sites are largely influenced by external stimuli such as atmospheric carbon dioxide, light, humans, animals and wind. Controlled laboratory experiments allow the study of mosquito behavior in the absence of external factors. Taxis boxes in field settings, on the other hand, provide a means to determine the orientation of mosquitoes in a more natural environment. The taxis boxes system permits the differentiation between mosquitoes' behavioral responses to experimental stimuli compared to natural stimuli that mosquitoes would regularly encounter in search of a host, such as human and animals odors, man-made structures, smoke and odor from cooking, and electrical or moon light [7]. In this experimental set up, a village approximately 70 meters east of the taxis boxes and a small settlement of homes with cattle along one side of the set up, only separated by a two meter high concrete wall, provided such conflicting external stimuli. However, we were able to measure mosquito responses to experimental stimuli despite the wealth of external stimuli. The treatment stimuli (coils and human) were placed downwind and taxis boxes were arranged in such a way that mosquitoes were placed upwind and could detect flowing smoke and host cues. The presence of light and human settlements in the east (downwind) likely increased the movement of mosquitoes to the chamber towards the stimuli, but this was controlled for in the analysis by the presence of the “no stimulus” control.

In the current study, taxis boxes were used to measure attraction inhibition and repellency of airborne pyrethroids. Here, Transfluthrin coils placed next to a human increased movement of mosquitoes within the taxis boxes. A higher proportion of mosquitoes left the middle chamber when a coil was next to a human. This indicated that Transfluthrin in combination with human volatiles increased activation and flight of mosquitoes, allowing them to leave the middle chamber. In contrast, compounds believed to be spatial repellents (e.g. linalool, dehydrolinalool and DEET) have been shown to slow down the flight and activity of mosquitoes and location of the host odor [33] hence acting as attraction inhibitors. Our study tested a pyrethroid, which has a different chemical structure compared to these spatial repellent compounds. This may explain the contrast in the response of mosquitoes observed in the current study. Exposure to pyrethroids is associated with high mosquito activity and flight, also referred to as excito-repellency [27]. Previous studies indicate that pyrethroid coils cause excitation and increased activity of mosquitoes [27].

Taxis boxes showed movement of mosquitoes towards the host despite the presence of coils. In fact, higher doses of Transfluthrin in coils increased the proportion of mosquitoes attracted to the human. Mosquitoes seen to fly towards humans even in the presence of coils indicated that airborne Transfluthrin does not prevent behavioral responses to attractive host cues. In contrast to these findings, some compounds including DEET, dehydrolinalool and linalool inhibit attraction to host odors [33], in particular to lactic acid [23].

It is possible that coils actually work at a later stage after detection and activation of host cues resulting in bite prevention [24]. Similar observations are reported elsewhere describing the effect of metofluthrin emanators and pyrethroid coils [24], [34], [35]. Catnip and 1-methylpiperazine acts at short distances to prevent mosquitoes from landing and biting humans but do not prevent attraction to attractive stimuli [36], [37]. This study reinforces the fact that airborne pyrethroids do not prevent attraction of mosquitoes to their hosts but likely interfere with the mosquito feeding process at the last stages after attraction to the host and prevent blood feeding. Other studies show that airborne pyrethroids exert multiple effects on a range of odorant receptors (ORs) and gustatory receptors located on antennae and feeding appendages of mosquitoes. They block, inhibit, or induce a number of different responses and scramble the host seeking process [22], [38], [39].

In this experimental design, mosquitoes were presented with conflicting stimuli: attraction to host odors versus the insecticide. A previous study showed that in such a case the need to feed can overpower the effect of the insecticide, hence the mosquito is still attracted to the host but is prevented from feeding [40]. This is evident with the use of insecticide treated bed nets, where mosquitoes are attracted to humans and attempt to feed through treated nets but then become irritated and move away without feeding [40].

A low proportion of mosquitoes (7% and 17%) moved away from the human even when there was a coil. This indicates that Transfluthrin coils did not induce movement away also known as taxis (repellency [20]). It may be speculated that Transfluthrin coils alone may have induced repellency by taxis of mosquitoes if experiments were conducted in the absence of the human. Unfortunately this was not directly measured in the current study because then the study would not have been representative of a natural setting (where coils are intended to be used in the presence of humans).

Therefore according to the results presented in this study, attraction inhibition, when mosquitoes are inhibited from responding to the host, was not induced by the presence of coils. On the other hand, coils induced one kind of repellency referred to orthokinesis or excito-repellency.

Coils used as a “point source” reduced bites by almost half when coils were placed 0.3 m away from the human and were effective even when the human was 20 m away from coils (Table 2). Interestingly the “bubble” was highly effective providing approximately 80% protection against bites when coils were 0.3 m away from the human (Table 3). Hence coils were more effective when used as a “bubble” rather than the “point source”. This highlights the need to consider presentation of the source of the active ingredient as a bubble around humans in order to achieve maximum efficacy. These results show the spatial activity and efficacy of volatile pyrethroids against mosquito bites. Efficacy of coils outdoors indicates that volatile pyrethroids may be an appropriate tool against outdoor biting mosquitoes and may be used outdoors in bars, restaurants, backyards or verandahs especially when multiple sources of repellent are used to ensure saturation of the space with active ingredient.

Previous studies indicate that mosquitoes inhibited by topical repellents from blood feeding are diverted to neighboring people who are not protected [41]. This may not be the case with volatile insecticides such as mosquito coils. This study shows that coils prevented bites when they were placed as far as 20 m away (Tables 2, 3), thus they provided area wide protection and hence likely extend protection to the non users at a particular distance from the source and reduce risk of diversion of mosquitoes. A study testing this hypothesis is currently being analyzed (Maia *pers. comm.*).

In addition to personal protection, this study also shows that mosquito coils offer temporal protection and hence are more likely to extend protection to non-users due to the prolonged feeding inhibition state. The aftermath of feeding inhibited mosquitoes was also investigated. In a closed laboratory setting (Peet Grady chamber), mosquitoes did not resume normal blood feed behavior up to 12 hours after they had been exposed to coils. We suggest that in addition to a spatial bubble, prolonged feeding inhibition may also protect non-users of coils to a certain extent, which would also reduce the risk of diversion. Similar results were reported in a study where the time of activation and flight of *Cx. quinquefasciatus*, *An. albimanus* and *Stegomyia aegypti* mosquitoes was reduced significantly 24 hours after they had been exposed to sublethal doses of Deltamethrin and Permethrin [42]. In the current study, mosquitoes resumed normal feeding after 24 hours. If mosquitoes miss one feeding opportunity due to exposure to coils, this is likely to prolong the gonotrophic cycle and may change the vectorial capacity of the mosquitoes [43].

However, when free-flying mosquitoes were exposed to coils under outdoor conditions in the SFT, there was no effect. This may be attributed to limited ventilation in the Peet Grady chambers resulting in reduced airflow accompanied by increased insecticide particles per area. This enabled mosquitoes to contact insecticides more easily, resulting in the large effect on blood feeding inhibition in mosquitoes exposed in the chambers. The effect of coils in the SFT was less pronounced probably due to the large surface area of the facility as well as natural airflow within the tunnel. It is hypothesize that sparse distribution of insecticide particles within the tunnel due to high airflow resulted in low concentration of insecticide particles. Therefore, mosquitoes did not contact sufficient insecticides in the SFT. It should be noted that coils used under outdoor conditions contained the standard dose of Transfluthrin (0.03%) meant for indoor use. It is therefore necessary to explore the effect of increasing the dose for products that are intended for outdoor use, in particular by advising users to put several coils around the area that they are occupying to create the “bubble effect”. In addition, there is need to determine the No observed effect level (NOEL) of airborne chemicals whilst in use outdoors.

The human biting rate of mosquitoes is one of the most important parameters that influences malaria transmission [3]. Hence, chemicals that interfere with feeding behavior of mosquitoes or prevent feeding altogether are likely to reduce transmission. This study emphasizes the importance of reduced blood feeding as the main indicator for efficacy of airborne pyrethroids used against outdoor biting mosquitoes.

## Conclusions

This study indicates that coils do not prevent attraction to the human nor induce taxis away from the human, but mainly prevent blood feeding. It is possible that pyrethroid based coils, specifically Transfluthrin, target receptors involved in feeding among other olfactory receptors. It is essential to conduct further studies to determine target sites of pyrethroid - based airborne particles in mosquitoes. This study provides critical information necessary for the development of target product profiles of spatial repellent products that can be used to complement existing mainstream malaria vector control tools.

Increased reports of outdoor biting and resting mosquitoes in endemic areas [44], [45] indicate that mainstream malaria control tools that target indoor biting and resting mosquitoes (LLINs and IRS) may not be sufficient to eliminate malaria especially when transmission occurs outdoors [46]. This study demonstrates the potential benefit of airborne pyrethroids for use against outdoor biting mosquitoes by reducing the outdoor man-biting rate, an important parameter of malaria transmission by providing personal protection through reduced mosquito bites (Tables 2, 3). It is worthwhile to conduct large scale clinical studies with entomological correlates of mosquito human-landing also observed to determine whether outdoor use of airborne insecticides in addition to the use of LLINs translates into additional protection from malaria, therefore complementing existing tools used against indoor biting and resting mosquitoes.
